# Combined pulmonary fibrosis and emphysema: a narrative review

**DOI:** 10.3389/fmed.2025.1683252

**Published:** 2025-12-04

**Authors:** Wenjing Zeng, Binmiao Liang, Xuemei Ou

**Affiliations:** Department of Pulmonary and Critical Care Medicine, West China Hospital, Sichuan University, Chengdu, China

**Keywords:** combined pulmonary fibrosis and emphysema, diagnosis, prognosis, complications, management

## Abstract

Combined pulmonary fibrosis and emphysema (CPFE) is defined as a clinical-radiological-physiological syndrome characterized by upper-lobe emphysema and fibrosis predominantly in the lower lobes. The diagnosis of CPFE remains challenging due to the opposing pathophysiological effects of emphysema and fibrosis, which can mask their characteristic clinical and imaging features. Although an international committee proposed standardized terminology for CPFE in 2022, uniform diagnostic criteria and optimal management strategies have not yet been established. Patients with CPFE exhibit reduced overall survival and higher mortality compared to those with chronic obstructive pulmonary disease (COPD) or idiopathic pulmonary fibrosis (IPF). This may be due to increased disease severity, as assessed by CT findings (the extent of fibrosis and emphysema), advanced age, and associated complications such as lung cancer, acute exacerbations, and pulmonary arterial hypertension. This narrative review synthesizes the literature on CPFE from 1990 to 2024, covering its historical background, epidemiology, pathogenesis, clinical presentation, diagnostic features (imaging and pulmonary function), disease course (diagnosis, prognosis, complications), and management.

## Introduction

Chronic obstructive pulmonary disease (COPD), emphysema, and interstitial lung disease (ILD) are the most prevalent chronic respiratory conditions. Nonetheless, it is becoming increasingly acknowledged that certain individuals may have both emphysema and ILD. This condition is known as a combination of pulmonary fibrosis and emphysema (CPFE). Originally described by Cottin et al., this condition features upper lung-predominant emphysema and lower lung-predominant fibrosis (1–4).

Although diagnostic criteria exist, the clinical identification of CPFE remains challenging. The challenge is exacerbated when assessing dyspneic patients, as CPFE-related anomalies may be obscured by comorbidities. Emphysema leads to a decrease in elastic recoil (i.e., increased compliance), whereas fibrosis results in enhanced elastic recoil (i.e.decreased compliance). When these opposing mechanisms balance each other, pulmonary function tests may show lung capacity and vital capacity measurements within normal ranges, thereby masking underlying severe pathology and leading to delayed or missed diagnosis (5).

.In this review, we summarize the clinical aspects of CPFE, including epidemiology, diagnostic features (including pulmonary fibrosis subtypes and functional assessment), characteristic imaging findings, treatments, complications, prognostic outcomes, and future directions.

### Pathogenesis and risk factors

**Figure 1:**
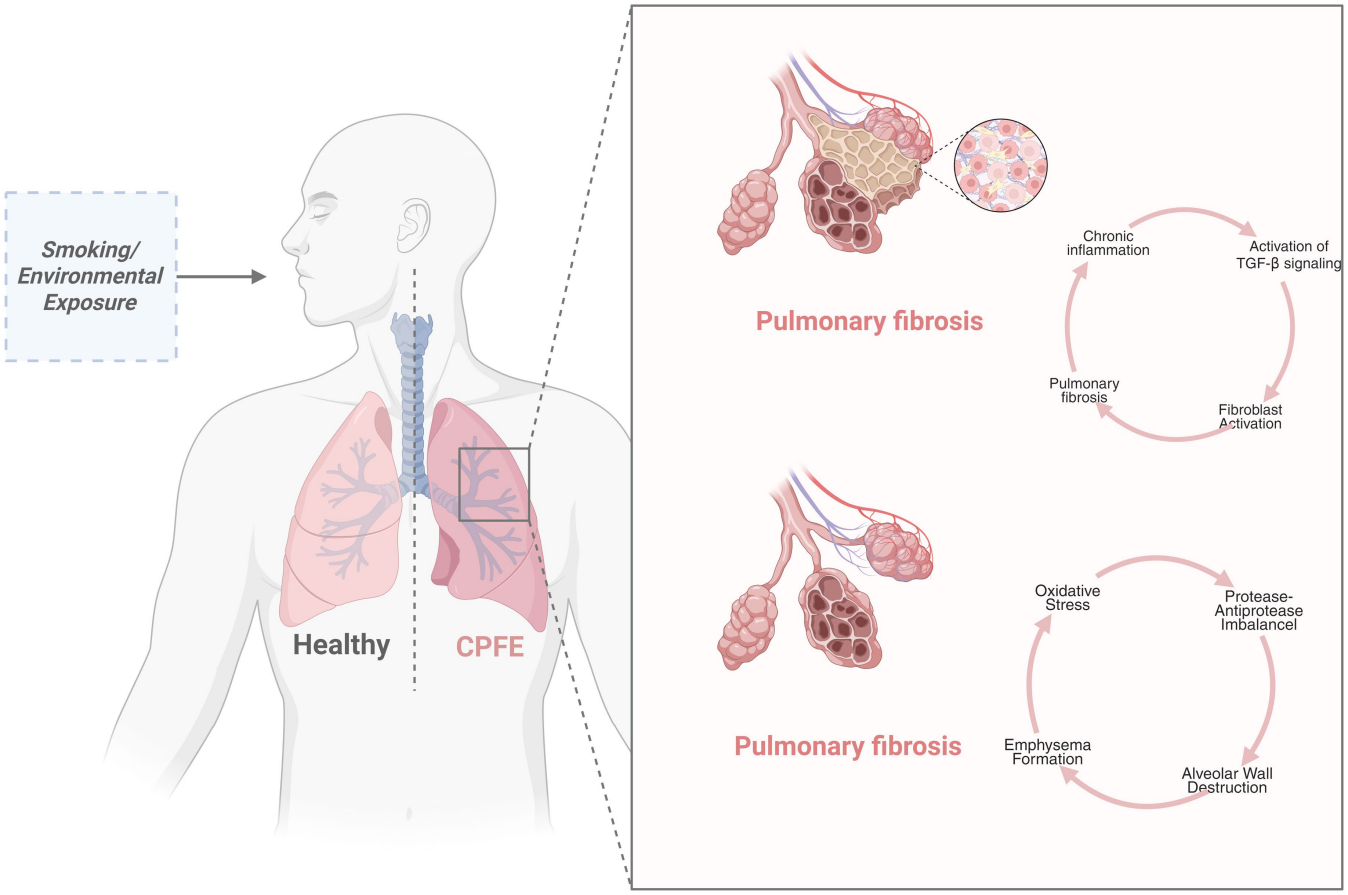
The pathogenesis of emphysema and pulmonary fibrosis is initiated by persistent damage to lung tissue as a result of smoking or environmental exposure. (1) In pulmonary emphysema, the core mechanism involves a protease–antiprotease imbalance. Activated macrophages and neutrophils release excess proteases (e.g., MMP-9, neutrophil elastase), degrading alveolar structural components such as elastic fibers and collagen, ultimately leading to irreversible airspace enlargement. (2) In pulmonary fibrosis, chronic inflammation promotes TGF-β-dependent Smad2/3 phosphorylation and non-Smad signaling through alveolar epithelial cell injury and M2 macrophage polarization. This cascade drives fibroblast-to-myofibroblast differentiation and excessive deposition of ECM proteins (e.g., collagen I/III), resulting in honeycomb-like fibrotic lesions. Created in BioRender. Zeng, W. (2025) https://biorender.com/lrhpgdf.

To date, the pathogenesis and pathophysiology of CPFE are unclear. Previous studies have shown that smoking is one of the risk factors for CPFE (6, 7). This exposure causes recurrent airway inflammation, facilitates immune complex accumulation in the lung interstitium, and disrupts inflammatory healing processes, cumulatively leading to pulmonary fibrosis. Concurrent small airway and alveolar epithelial damage from smoking triggers emphysematous changes, resulting in combined pulmonary fibrosis and emphysema (Figure 1) (8–13). Long-term occupational exposure to asbestos, coal dust, talcum powder, trichloroethylene, pesticides, or welding fumes is associated with an elevated risk for CPFE development (14, 15). Furthermore, CPFE development is acknowledged in nonsmoking populations, especially among those with connective tissue diseases (CTD). Ariani et al. identified CPFE in 43 out of 470 individuals with systemic sclerosis by high-resolution computed tomography (HRCT) of the chest. A multicenter investigation demonstrated markedly increased serum antinuclear antibody (ANA) levels in patients with CPFE relative to controls with idiopathic pulmonary fibrosis (IPF).

Additionally, a recent population-based cohort analysis further revealed elevated CPFE prevalence among lung cancer patients, particularly those with comorbid rheumatoid arthritis (RA) (16–20). Together, these findings indicate that CTD may act as both a risk factor and a pathogenic component in CPFE; however, the link between CTD and CPFE remains inadequately defined. Significant knowledge gaps remain regarding the clinical characteristics, prognosis, and heterogeneity across CTD subtypes in the CPFE-CTD overlap syndrome. Therefore, focused mechanistic research and clinical validation studies are essential to elucidate the underlying pathophysiological mechanisms.

Emerging evidence indicates that genetic factors also contribute to the development of CPFE.

Several studies have implicated specific genetic factors, including peptidase D (PEPD), telomerase reverse transcriptase (TERT), surfactant protein C (SFTPC), and adenosine triphosphate-binding cassette subfamily A member 3 (ABCA3) gene mutations (21–24). Telomeres, nucleoprotein complexes located at the termini of eukaryotic chromosomes, inhibit the activation of the DNA damage response at chromosomal ends (25, 26). Telomere dysfunction triggers DNA damage signaling, leading to p53 activation and culminating in cellular senescence and apoptosis. Persistent p53 activation during progressive telomere shortening exacerbates tissue-specific pathologies, including pulmonary fibrosis, aplastic anemia, and cirrhotic liver disease (27). Through Mendelian randomization analysis, Anna et al. established a causal link between telomere attrition and IPF, particularly in patients with co-existing IPF and COPD. In animal models exposed to cigarette smoke and bleomycin, mice with telomere shortening and dysfunction exhibited increased incidence and severity of both pulmonary emphysema and fibrosis.

Overall, CPFE pathogenesis involves a multifactorial interplay of environmental exposures and genetic determinants (28, 29).

### Clinical features

**Figure 2:**
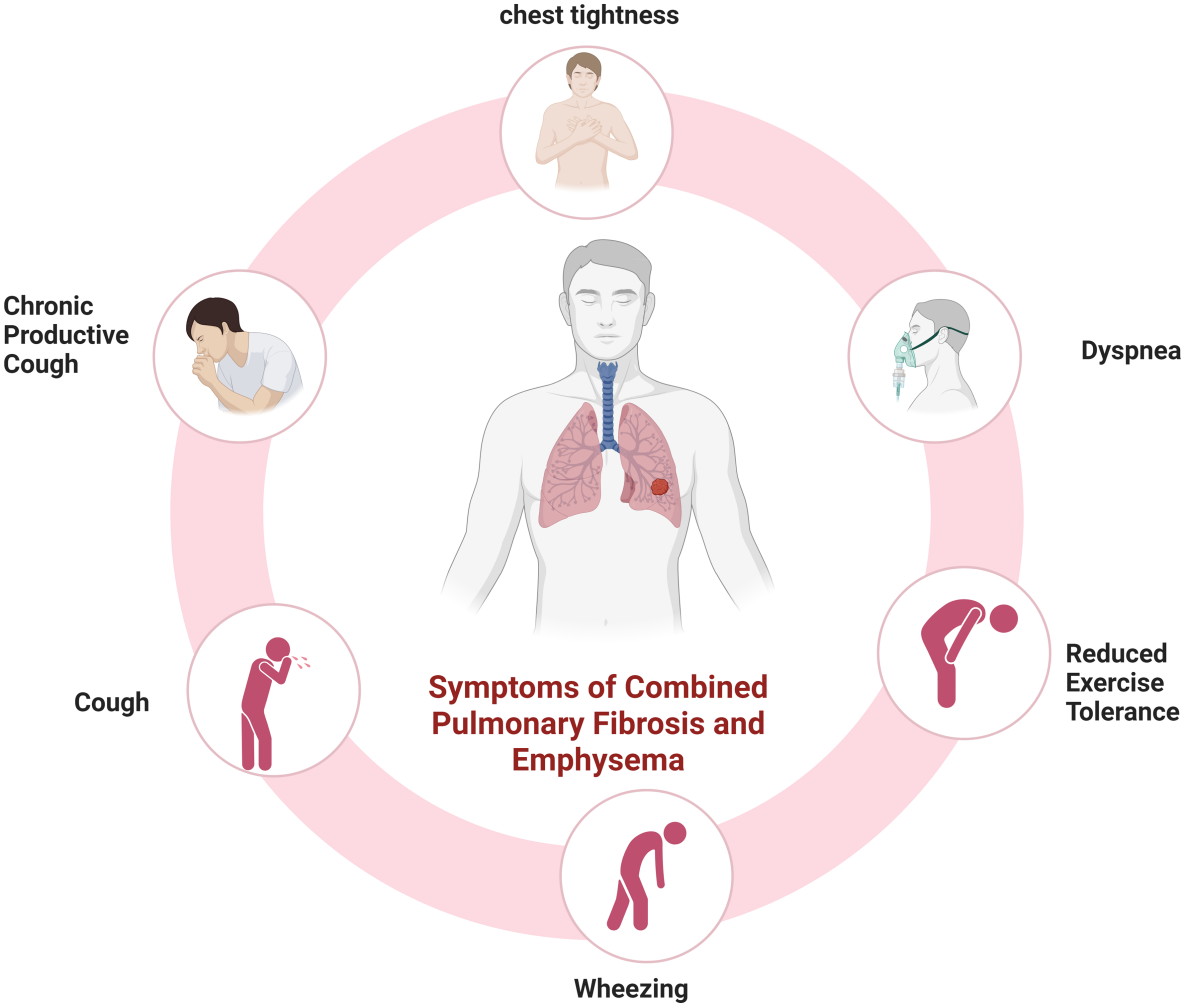
Common clinical manifestations in patients with CPFE. Created in BioRender. Zeng, W. (2025) https://BioRender.com/7szy1o8.

Patients with CPFE are predominantly male smokers aged 60–80 years, who typically present with gradually progressive exertional dyspnea and a persistent chronic cough. Common clinical manifestations are presented in Figure 2. On physical examination, bibasilar fine inspiratory crackles are commonly observed, along with diminished breath sounds in the upper lung zones. Digital clubbing may also be present in some cases (2, 14, 30, 31).

### Diagnostic testing

**Figure 3:**
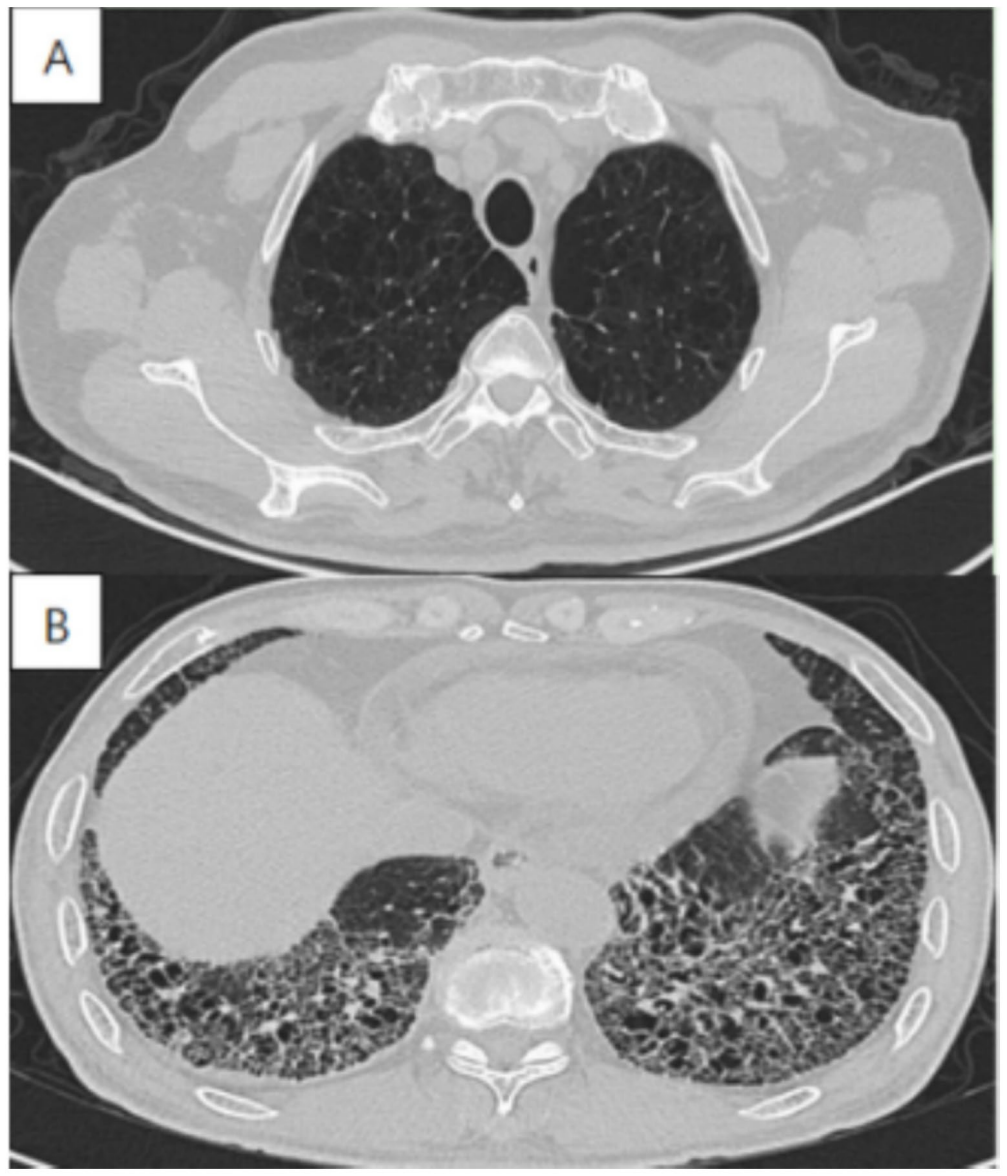
High-resolution computed tomography in a 72-year-old male with CPFE, showing **(A)** paraseptal emphysema in the upper lobe, **(B)** lower-zone-predominant fibrosis.

Imaging in CPFE reveals a characteristic dual pattern of upper-lobe emphysema and lower-lobe interstitial fibrosis (Figure 3). The emphysematous alterations in the upper lobes primarily consist of centrilobular and paraseptal bullae, exhibiting a distinct distribution pattern relative to pure COPD. Whereas centrilobular emphysema is predominant in COPD patients, paraseptal involvement is observed in approximately two-thirds of CPFE patients—a signature feature of this syndrome (Table 1) (14, 32, 33). While emphysema and fibrosis are usually distinguishable on HRCT, their differentiation can be challenging during early disease stages or when both pathologies are diffusely distributed throughout the lungs. Hyperinflation leads to reduced pulmonary vascular markings and diaphragmatic flattening, which are hallmark radiographic features of advanced emphysema. Conversely, advancing fibrosis reduces the lung volume, reversing emphysema-associated overinflation. Mori et al. reported that, in comparison with patients with COPD alone, CPFE patients had considerably elevated KL-6 levels (20). These findings suggest that KL-6 has potential as a practical screening indicator for CPFE. Therefore, future studies should establish validated biomarkers for early CPFE detection and develop tools to discriminate fibrotic from emphysematous components.

**Table 1:** Imaging characteristics of pulmonary fibrosis and emphysema.

Component	Examples	Remark
Fibrosis	UIP	The most common type of fibrosis in CPFE
	Fibrotic NSIP	
	Desquamative interstitial pneumonia	
	Unclassifiable fILD	
	AEF, SRIF, Etc.	
Emphysema	Centrilobular	The most common emphysema type in CPFE
	Paraseptal	
	Mixed (paraseptal and centrilobular)	
	Admixed (with fibrosis)	
	Thick-walled large cysts	
	Emphysema, pattern not specified.	

UIP, usual interstitial pneumonia; NSIP, nonspecific interstitial pneumonia; fILD, fibrosing interstitial lung disease; AEF, airspace enlargement with fibrosis; SRIF, smoking-related interstitial fibrosis.

CPFE is characterized by distinct pulmonary function features that differentiate it from either emphysema or IPF alone. Ventilatory parameters—such as FVC, FEV₁—typically remain normal or show only mild impairment, whereas diffusing capacity for carbon monoxide (DLCO) is frequently severely reduced. Certain patients display isolated DLCO impairment; concurrently, emphysema evolves via elastic recoil deficiency-induced compliance elevation, resulting in airway collapse and alveolar dilation (3, 30, 34, 35). Conversely, pulmonary fibrosis increases elastic recoil and decreases compliance, thereby maintaining airway patency through traction. When these opposing effects counterbalance each other, patients with CPFE may exhibit preserved lung volumes and vital capacity, potentially resulting in delayed diagnosis.

### Diagnosis

Currently, there are no universally accepted diagnostic criteria for CPFE (2). In practice, some studies define CPFE by quantifying emphysema severity, employing diagnostic thresholds of ≥10% or ≥20% emphysema concurrently with ≥10% or ≥15% fibrosis (Table 2) (36–40). The most authoritative consensus to date, which was jointly issued in 2022 by the American Thoracic Society (ATS), the European Respiratory Society (ERS), and the Japanese Respiratory Society, now serves as the widely recognized clinical guideline for the CPFE (41).

**Table 2:** Diagnostic criteria for CPFE across different temporal periods.

Phase	The subsequent phase of research (2005–2022)	Quantitative Imaging Thresholds	Landmark Literature
Initial conceptualization Phase(2005)	Presence of emphysema on CT scan, defined as well-demarcated areas of decreased attenuation in comparison with contiguous normal lung and marginated by a very thin (1 mm) or no wall, and/or multiple bullae (0.1 cm) with upper zone predom0inance. Presence of a diffuse parenchymal lung disease with significant pulmonary fibrosis on CT scan, defined as reticular opacities with peripheral and basal predominance, honeycombing, architectural distortion, and/or traction bronchiectasis or bronchiolectasis; focal ground-glass opacities and/or areas of alveolar condensation may be associated but should not be prominent. Patients with connective tissue disease at the time of the diagnosis of CFPE were excluded from the study, as well as patients with a diagnosis of other interstitial lung diseases, such as drug-induced interstitial lung disease, pneumoconiosis, hypersensitivity pneumonitis, sarcoidosis, pulmonary histiocytosis, lymphangioleiomyomatosis, and eosinophilic pneumonia.	Absence of a clearly defined quantitative threshold	Cottin et al. (2)
The subsequent research stage (2005–2022)	Quantitative-threshold approach: ① Emphysema: ≥10% or ≥20% lung volume ② Fibrosis extent: ≥10% or ≥15% Qualitative-morphology approach: ① Diagnosis based solely on morphological characteristics (e.g., honeycombing)	Diagnostic thresholds adopted: ① Emphysema: LAA-950 > 10% ② Pulmonary fibrosis >15% involvement of total lung volume	Kim et al. (70); Jacob et al. (12)
The standardized consensus development process (2022)	Requires concurrent presence of both: Emphysema: Well-defined areas of low attenuation bounded by a wall ≤1 mm or no wall, involving ≥5% of total lung volume. Pulmonary Fibrosis: Any subtype of pulmonary fibrosis Inclusion requires fulfillment of the CPFE criteria along with at least one of the following: Emphysema accounts for ≥15% of the total lung volume. The characteristic physiologic profile is one of preserved lung volumes and airflow but a severely or disproportionately impaired DLco, a pattern that is particularly indicative in the absence of extensive HRCT abnormalities or pulmonary hypertension Precapillary pulmonary hypertension that is not attributable solely to emphysema (FEV₁ >60%), pulmonary fibrosis (FVC >70%), or another identifiable etiology	—	Cottin et al. (41)

Future large-scale, multicenter cohort studies are needed to establish critical thresholds for emphysema and pulmonary fibrosis in CPFE, such as AI-driven automated lesion segmentation combined with HRCT density histograms and airway parameter analysis. To guide targeted interventions, CPFE patients are categorized based on the combined severity of emphysema and fibrosis. In those with predominant emphysema, inhaled pharmacotherapy forms the mainstay of treatment, whereas patients with predominant fibrosis receive combination antifibrotic therapy.

Furthermore, additional research is necessary to investigate blood biomarkers(such as KL-6 and SP-D) for objective quantification of lesion extent. Such efforts should also aim to develop a multidimensional CPFE staging system that incorporates imaging, functional, and clinical parameters, and to create a phenotype-based individualized treatment decision tree for evidence-based targeted interventions.

### Prognosis and complications

Previous research has demonstrated that the median survival of CPFE patients varies from 0.9 to 8.5 years (33, 42). Prognostic determinants include age, CT-defined disease severity (extent of fibrosis and emphysema), and major comorbidities such as lung cancer (LC), acute exacerbation(AE), and pulmonary arterial hypertension (PAH) (41).

PAH is present in 47–90% of CPFE patients, with a mean sPAP of 41.9 ± 19.7 mmHg (43–45). Sugino et al.’s echocardiographic sPAP analysis demonstrated greater annual pulmonary arterial pressure progression in CPFE than in IPF. Patients with CPFE and PAH exhibit a worse prognosis than those with CPFE or IPF alone, with a 1-year survival rate of approximately 60%, as reported in previous studies (40, 46).

LC is also a common complication in patients with CPFE, primarily affecting elderly male smokers. The lesions typically occur in the lower lobes of the lungs, often in areas of pulmonary fibrosis. The predominant pathological subtypes are adenocarcinoma and squamous cell carcinoma (47–52). Upon diagnosis of lung cancer, patients with CPFE often present with advanced-stage disease. Owing to nonspecific clinical symptoms, detection and diagnosis are frequently delayed. More significantly, the tumor may be obscured by underlying pulmonary fibrosis and emphysema, complicating timely detection via chest CT scans and potentially leading to misdiagnosis or delayed diagnosis (53, 54). Both emphysema and pulmonary fibrosis independently increase the risk of LC in patients with CPFE. Multiple studies confirm a significantly elevated risk of malignancy compared to either condition alone (38, 40, 55–57). Additionally, smoking, family history of cancer, body mass index (BMI), fibrinogen levels, and serum C3 levels are independently associated with LC development in patients with CPFE (54, 58). Compared with IPF-LC, COPD-LC, or CPFE alone, patients with CPFE-LC exhibit reduced overall survival (30, 48, 59–61). This poor prognosis may be associated with the severity and type of pulmonary fibrosis, the emphysema phenotype, the stage of lung cancer, and the implementation of treatment strategies.

Current evidence indicates that the following factors may serve as predictors of prognosis in CPFE-LC patients: lung function indicators (e.g., carbon monoxide diffusion capacity, forced expiratory volume in the first second and forced vital capacity), disease severity (e.g., the extent of pulmonary fibrosis or emphysema and TNM staging of lung cancer), AE, the composite physiologic index and higher normal lung scores (62–68).

AE critically increase CPFE mortality risk and are potentially associated with COPD or ILD. Lee et al. found that pulmonary infection was the main cause of AE in patients with CPFE. Environmental factors, such as air pollution and smoking, represent established risk factors for AE (69–71). Notably, loxoprofen, a nonsteroidal anti-inflammatory drug, has been implicated in triggering exacerbations of CPFE (72). Current evidence remains limited, but decreased FVC, DLCO, LC, and elevated GAP scores constitute established predictors of AE (73, 74). In addition, it is unclear whether the incidence of AE-COPD in CPFE patients is consistent with that in patients with single-disease COPD.

### Treatment

Currently, there is no effective treatment for CPFE. Evidence supports the benefits of smoking cessation, oxygen therapy, pulmonary rehabilitation, and vaccination in the management of CPFE (41). Although the efficacy of pulmonary rehabilitation has not been definitively established in CPFE, it is nevertheless recommended for most patients due to its potential to improve exertional dyspnea and quality of life. Simone et al. demonstrated that moderate-intensity aerobic exercise combined with breathing training for 4 weeks significantly improved physical fitness, quality of life, and mood in rehabilitation patients. However, pulmonary function showed no significant improvement following this intervention (75, 76). Currently, high-quality studies evaluating the efficacy of pulmonary rehabilitation in CPFE remain limited. In the future, more research should focus on developing individualized exercise rehabilitation regimens for patients with CPFE. Oxygen therapy should be initiated in patients with hypoxemia at rest or during exertion. Long-term oxygen therapy may prevent complications of chronic hypoxemia (77). Infection is a significant contributing factor to the progression of both COPD and pulmonary fibrosis. Clinical guidelines accordingly advise vaccination in affected individuals. For patients with CPFE, appropriate vaccinations (such as influenza, novel coronavirus, and pneumococcal vaccines) are recommended to prevent infection, decrease the frequency of AE, and slow disease progression (78).

Currently, no targeted pharmacological therapies are available for CPFE. However, due to the inherent heterogeneity of interstitial lung diseases and the frequent coexistence of emphysema, separate management of pulmonary fibrosis and emphysema is warranted. Treatment selection for CPFE depends on whether the disease is predominantly inflammatory or fibrotic, guiding the use of anti-inflammatory or antifibrotic agents. For patients with CPFE who have progressive fibrosis, antifibrotic agents such as Nintedanib or pirfenidone may be considered. However, the therapeutic efficacy of antifibrotic agents in CPFE remains inconsistent. One study employed ultrasound imaging of lung fissures to assess pirfenidone, demonstrating limited therapeutic benefit in CPFE (79–81). By contrast, a phase III clinical trial investigated the efficacy of Nintedanib in progressive pulmonary fibrosis and showed that this agent can slow disease progression. A separate study compared antifibrotic agents (including Nintedanib and pirfenidone) in patients with IPF and CPFE. This investigation found that long-term use (≥12 months) of these agents was associated with improved survival in both groups.

Evidence supports the use of bronchodilators in CPFE patients with reversible airflow obstruction. However, their efficacy in those without airflow limitation remains poorly established. Dong et al. demonstrated that ICS/LABA therapy improved pulmonary function and reduced both the frequency and severity of acute exacerbations in patients with CPFE (82). However, these findings do not establish a therapeutic benefit of bronchodilators in CPFE, as the study was constrained by a small sample size (45 patients receiving bronchodilators vs. 24 patients not receiving bronchodilator therapy), non-randomized design with potential selection bias, and a lack of adjustment for disease severity. Lung transplantation remains the only life-extending treatment for patients with CPFE and represents the preferred intervention for those with end-stage disease. In a large-scale study, Takahashi et al. reported a 5-year post-transplant survival rate of 79% in CPFE patients (14, 44, 83).

In summary, CPFE arises from synergistic interactions between the pathological processes of pulmonary fibrosis and emphysema. Clinical management remains challenging and is guided by the relative extent, subtypes, and severity of these two components. Inhaled bronchodilator therapy should be considered for patients with CPFE and significant airflow obstruction. Antifibrotic therapy may be an option for CPFE patients with progressive pulmonary fibrosis. Inhaled bronchodilator therapy should be considered for patients with CPFE and significant airflow obstruction. For those with progressive pulmonary fibrosis, antifibrotic therapy may represent an option. However, the need for and modality of treatment in patients without significant airflow limitation or progressive fibrosis remain unclear and require further investigation. Prospective, large-scale clinical trials are necessary to evaluate the efficacy of antifibrotic agents and bronchodilators in specific CPFE subtypes. Lung transplantation is the only intervention proven to prolong survival in end-stage CPFE.

## Conclusion

CPFE is a clinical imaging syndrome. It is characterized by the interaction (not simply superimposition) of pulmonary fibrosis and emphysema. Owing to the interference and mutual masking of the imaging characteristics of pulmonary emphysema and early pulmonary fibrosis on HRCT, early identification is limited. Therefore, future research should employ AI-driven quantitative imaging analysis integrated with multimodal biomarkers and clinical data to achieve early detection, precise phenotyping, and personalized treatment. Currently, no targeted therapies are available for CPFE; management focuses on supportive measures such as smoking cessation, vaccination, oxygen therapy, and treatment of comorbidities. Advancing targeted phase II/III clinical trials and establishing integrated imaging-biomarker-clinical phenotype prognostic models are imperative to optimize individualized treatment strategies and improve quality of life.
